# Editorial: Sensory Processing Across the Lifespan: A 25-Year Initiative to Understand Neurophysiology, Behaviors, and Treatment Effectiveness for Sensory Processing

**DOI:** 10.3389/fnint.2021.652218

**Published:** 2021-04-09

**Authors:** Lucy Jane Miller, Elysa J. Marco, Robyn C. Chu, Stephen Camarata

**Affiliations:** ^1^Department of Pediatrics (Emeritus), University of Colorado, Denver, CO, United States; ^2^Sensory Therapies and Research Institute for Sensory Processing Disorder, Centennial, CO, United States; ^3^Cortica (United States), San Diego, CA, United States; ^4^Radiology & Biomedical Imaging, University of California San Francisco, San Francisco, CA, United States; ^5^Growing Healthy Children Therapy Services, Rescue, CA, United States; ^6^School of Medicine, Vanderbilt University, Nashville, TN, United States

**Keywords:** sensory processing, sensory integration, neurophysiology of sensation, sensory phenotype, treatment effectiveness

## Growing Scientific Knowledge in Sensory Processing

### The Growth of Science

Science must evolve. Kuhn (1970) proposed that scholars adapt their research as new information is discovered and described how the growth of knowledge results in paradigm shifts. Science advances in increments based in part on fact, law, and theory and in part on imagination, hypothesis, and error. The articles in this issue demonstrate Kuhn's premise and the need for rigorous, multidisciplinary, empirical research to underlie a new field such as sensory processing.

### Brief History of SPD

Ayres (1972) was the first to explore sensory processing, focusing on children with learning disorders. In impressive detail, she collected and studied clinical observations, standardized assessment data, and treatment methods. She proposed a new syndrome, which she termed “sensory integration dysfunction” (SID). From 1964 to 1966, Ayres, an occupational therapist, conducted post-doctoral studies at UCLA Brain Research Institute. Membership in the neuroscience department permitted Dr. Ayres to learn the culture of research in a transdisciplinary environment and to hypothesize the brain/behavior connection in the newly conceptualized condition.

This issue of *Frontiers* celebrates the growth of scientific knowledge, founded upon Ayres' research from 1960 to 1988 (Ayres, 1955, 1964, 1966a,b; Ayres, 1971, 1977, 1989) and progressing with the support of a 25-year initiative, by the Wallace Research Foundation (WRF) (1994–2019). The WRF funded over 50 scholars, who, as members of the SPD Workgroup, worked for two decades to evaluate whether the reliability and validity of sensory processing issues were strong enough to suggest a new syndrome, which was termed Sensory Processing Disorder (SPD).

### Breadth of Knowledge Gained

Articles in this issue of **Frontiers** represent many of the latest studies in SPD research and represent the ripple effect as discussed in Kuhn's premise; that is, Ayres' early work led to the science conducted by the WRF SPD workgroup and many other important researchers in the field. We review the scientific breakthroughs that have occurred in the past quarter-century and propose a theoretical model that may be helpful to future researchers trying to specify the reliability and validity of SPD as a new syndrome (Pennington, 1991). While not exhaustive of the work in sensory processing done by the neuroscience and occupational therapy communities as a whole, the framework below represents the five areas in Pennington's model of syndrome validation. For the interested reader, we have included multiple citations for publications funded by the WRF below.

#### Etiology and Epidemiology

Ahn et al. (2004), reported that 13% of kindergarten parents indicated significant sensory challenges. As this was derived from a 40% response rate, the estimate of SPD prevalence in a community sample was 5%. Ben-Sasson and Carter et al. (Ben-Sasson et al., 2009, 2010; Carter et al., 2011), furthered this inquiry by assessing SPD in a 10-year, prospective study of all births in New Haven, Connecticut. In children up to 8 years of age, 16% had symptoms of SPD, with 75% reporting no additional mental health diagnosis. In addition, Goldsmith et al. (Keuler et al., 2011; Van Hulle et al., 2012, 2015, 2018, 2019), conducted elegant twin studies concluding that sensory symptoms occur significantly more often in identical than in fraternal twins, implicating a genetic link. These studies were pivotal in shaping the landscape of this condition and highlighting the need for further etiological studies.

#### Pathogenesis

Over the past two decades, we have furthered our understanding of the neurophysiology of sensory processing in general, however additional research is needed for specific subtypes of the condition. For example, Miller et al. (McIntosh et al., 1999; Miller et al., 1999) contributed that children with SPD show increased electrodermal responses and decreased habituation while Davies and Gavin (Davies and Gavin, 2007; Brett-Green et al., 2008; Davies et al., 2009, 2010; Gavin et al., 2011; Chang et al., 2012; Brett et al., 2016; Lagasse et al., 2019; Crasta et al.) found evidence of reduced sensory gating. Utilizing rodent models to better understand the mechanisms of these electrophysiologic differences, Bauman, Levin and colleagues (Levin et al., 2005, 2007; Schmajuk et al., 2006, 2009; Roegge et al., 2007; Larrauri and Levin, 2012; Mahendra et al., 2012; Skefos et al., 2014; Larrauri et al., 2015; McMahon et al.) determined that sensory gating deficits were related to activity of cholinergic, glutamatergic, and adrenergic receptors which suggests potential therapeutic approaches.

Animal studies have also greatly contributed over time to our understanding of the interplay of information among the individual sensory streams and multisensory integration. For over 20 years, Schneider et al. (Schneider et al., 1991, 2007, 2008, 2009, 2011, 2013, 2017; Moore et al., 2008; Coe et al., 2010; Converse et al., 2013; Schneider et al.), studied Rhesus monkeys. With positron emission tomography (PET) imaging, Schneider's findings suggest that SPD affects dopamine (DA) pathways, resulting in decreased regulation of sensory and affective processes and increased over-responsivity to stimuli. Stein and Rowland et al. (Stein, 1998, 2012; Fuentes-Santamaria et al., 2008, 2009; Stein et al., 2009; Yu et al., 2009, 2010; Cuppini et al., 2010, 2018; Rowland et al., 2014; Xu et al., 2015; Miller et al., 2017), have informed the field regarding the importance of the superior colliculi as a multisensory integrating region. In feline models, they found that simultaneous auditory-visual exposure radically changes input to neurons, honing the cat's ability to detect, identify, and respond to environmental events.

This understanding was then applied to children with autism and SPD by Molholm and Foxe who show that children with sensory over-responsivity have reduced auditory-visual integration affecting their perception of speech in noisy environments. Marco and Mukherjee's (Marco et al., 2011, 2012, 2018; Owen et al., 2013; Wickremasinghe et al., 2013; Mukherjee et al., 2014; Chang et al., 2016; Demopoulos et al., 2017; Brandes-Aitken et al., 2019; Payabvash et al., 2019; Tavassoli et al., 2019) structural neuroimaging work revealed that children with SPD show decreased white matter connectivity predominantly in the posterior brain regions that correlates with sensory function and has elements that are overlapping and some that are distinct from an autism cohort. They also show that there is a significant overlap in visual motor control and cognitive control deficits in children with SPD which result from disruption of shared white matter tracts (Brandes-Aitken et al., 2018, 2019). Additional work by Marco and Nagarajan et al. (Demopoulos et al., 2017), using magnetoencephalographic functional imaging suggests that children with SPD show an intermediate phenotype with regard to the time course of somatosensory (tactile) processing relative to children with autism spectrum disorders (ASD) and neurotypical controls.

#### Phenotype

Phenotype (core and secondary symptoms) exploration of sensory over-responsivity, also termed hyper-reactivity or sensitivity, has been researched in otherwise neurotypical individuals or in cohorts with additional mental health conditions (Miller et al., 2009; Schoen et al., 2009, 2014a; Tavassoli et al., 2018). Cermak et al. (Zobel-Lachiusa et al., 2015; Bar-Shalita and Cermak, 2016; Chistol et al., 2018; Ben-Sasson et al., 2019; Kilroy et al., 2019), measures aversive sensory responsiveness in individuals with autism and in the general population, concluding that sensory responsiveness has high correlation to pain perception. Ben-Sasson et al. suggest that slow sensory habituation may underlie over-responsivity in individuals with obsessive compulsive disorder. In addition to electroencephalographic studies cited above, Gavin and Davies discuss attention and sensory profiles in children with SPD and ASD. They successfully categorize 76.8% of participants (SPD vs. ASD vs. Typical) for group membership based on standardized test scores.

There are various assessment tools utilized for determining the extent of sensory processing dysfunction, with parent report measures, the Sensory Profile 2 and Sensory Processing Measure (Diane Parham et al., 2007; Winnie Dunn, 2014), being the most commonly used in research, clinics, and schools. There are excellent reviews for more in depth coverage of this important topic (Eeles et al., 2013; Yeung and Thomacos, 2020). None of the current assessments evaluate all domains thought to be related to sensory processing. The development of a standardized direct assessment tool with psychometric data for multiple facets of sensory processing (sensory modulation, sensory-based motor, and sensory discrimination) is important to future research and clinical phenotyping. The Sensory Processing Three Dimensions Measure (SP3D) (Lane et al., 2000; Miller and Lane, 2000; Miller et al., 2001, 2007a; Schoen et al., 2008, 2014b, 2017) is one of the assessments being developed to fill the need since the previous standardized scale (Ayres, 1989) is no longer published. Miller, Schoen and Mulligan (Miller et al., 2020a) are completing national standardization of the SP3D and Schoen et al. (Schoen et al., 2008, 2014b, 2017; Mulligan et al., 2019a,b), have contributed articles on this topic. Future research will use these comprehensive assessments to connect the phenotypic information to neuroimaging, leading to deeper understanding of sensory processing. Moreover, there is an ongoing need to further develop the phenotype and to specify the unique and shared features with other established phenotypes (e.g., ASD, Developmental Language Disorder, see Skuse, 2000). The continued refinement (and precision) for an SPD phenotype (or phenotypes as the data may ultimately show), is foundational for advancing the knowledge base to inform future behavioral, genetic, neurological and treatment research.

#### Treatment Effectiveness

Miller et al. (2007b), conducted a pilot randomized controlled trial (RCT) of treatment using occupational therapy for children with sensory modulation disorder and found improvement in personalized goals, attention and social function based on the Leiter International Performance Scale-revised. Schoen et al. (2018), reports sensorimotor and adaptive function improvement based on a chart review of 179 children receiving occupational therapy. Miller et al. (Miller et al., 2018, 2020b; Schoen et al., 2019), discuss a comprehensive new treatment based on these findings and clinical observations, which uses a sensory and relationship-based approach, the STAR Frame of Reference^©^. Pfeiffer et al. (2011) compared fine motor treatment vs. sensory integration (SI) therapy for children with ASD and showed additional benefit from SI for autism mannerisms and personalized goals. Similarly, Schaaf et al. (2014) assessed children with autism comparing SI with “usual care” and reported benefits for personalized goals, self-care and socialization. In this journal, Camarata and colleagues review the sensory integration treatment and other treatment issues including: (1) clinical trials and methods used in applied behavior analysis, (2) the neural-scientific paradigm of multisensory processing, and (3) controlling for potential confounds (Camarata, 2014a,b; Stevenson et al., 2014a,b,c; Davis et al., 2015; Stevenson et al., 2016). Additionally, a WRF-funded project investigating the role of brain training for cognitive control in children with SPD has contributed to the first digital therapeutic device for attention (EndeavorRX) being approved by the United States FDA (Anguera et al., 2017).

#### Developmental Course

There is a dearth of longitudinal work investigating sensory processing, including multisensory integration, across early development and with aging. McKibbon et al., study the trajectory of SPD by examining a sample of 231 adults who had emotion regulation difficulties that were preceded by SPD in childhood. This study opened up examination of the developmental trajectory of SPD, suggesting that SPD has a childhood onset and discussing possible mechanisms that might be involved in the progression. Concluded was that childhood SPD predicts Anxiety Disorder in adults defined by difficulties with emotion regulation, mediated by adult SPD symptoms. The article by Tavassoli et al. (2014) looking at sensory symptoms in adults with SPD and Autism further explores this hypothesis. Ben-Sasson et al. (2010) followed 521 children from infancy to 8 years old and concluded that early sensory sensitivities were associated with sensory over responsivity at school age. Ben-Sasson et al. (2019) recently completed a meta-analysis of sensory symptoms in children with ASD throughout the lifespan, with various studies with findings that sensory symptoms can increase, decrease, or be stable throughout the lifespan and calling for additional research to look more in depth at the moderating effects of age.

### Value of Empirical Data for Change and Future Research

When defining a new science, reliable quantitative benchmarks often do not exist as was true with SPD. Thus, continuing definition is required. Conceptualization of the field grows as empirical data is obtained. This results in changes in theories and affects practice significantly. With the WRF initiative and world-wide study, each project increased knowledge incrementally, and the collaborative effect overall substantially expanded the understanding of SPD as a brain-based disorder.

Finding that the binary conception of SPD as a “disorder” was too simple, we view SPD not as a singular entity, but rather, a continuum of function-to-dysfunction for any given individual, which indicates a “dimension” rather than a “disorder”).

In the next quarter century, research will question brain networks, neurochemistry, and neural firing that explains the facets of disrupted sensory processing, from:

- Low-level abilities *(perceive, protect, and react)* to
- Mid-level processing *(integrate, process, and relay)* to
- High-level function *(discriminate, plan, and respond)*.

With time and increased knowledge, adaptations to terminology have occurred. Naming SPD and categorizing the symptoms have validated parents' concerns and are partially related to including sensory hypo and hyper-reactivity as clinical constituents of autism spectrum disorders in the *Diagnostic and Statistical Manual-5*. Extensive research establishing the groundwork for additional studies has been accomplished and has provided a foundation for understanding that SPD is prevalent, diagnosable, and treatable. The WRF initiative, which we applaud and celebrate, has encouraged us to “keep in mind what is assumption and what is fact … [for] truth like infinity is to be forever approached, but never reached” (Ayres, abid, p. 4).

## Author Contributions

All authors significantly contributed to the conception, data acquisition, drafting, and revision of the article.

## Conflict of Interest

LM was the former director of the STAR Institute in Greenwood Village, CO. Much of the research described in the special issue was conducted while she was the director of the center and was compensated in that role. The center provides workshops, in-service training and direct client services for children with disabilities and their families. In addition, LM is an author on the Sensory Processing 3 Dimensions (SP3D), a commercially available assessment and as such, will receive royalties from Western Psychological Services, which will publish the test once data collection is finished and the examiner's manual is written (possibly 2024, depending when the COVID crisis is managed and testing can begin again). LM also receives royalties as the author of Sensational Kids, published by Penguin: NYC. Finally, LM was the awarded funding from the Wallace Research Foundation, which supported much of the research reported in this Frontiers special issue. None of the compensation was contingent upon any specific outcomes or findings from the research reported herein nor, for that matter, in any of LM research publications. None of the articles in this issue directly promotes any specific approach or product. EM is the executive director of neurodevelopmental medicine at Cortica Healthcare in San Rafael, CA where she provides clinical care, conducts research, and oversees the neurodevelopmental medicine faculty and support. She has received research funding from the Sensory Neurodevelopment and Autism Program crowdfunding campaign, UCSF RAP awards, the Wallace Research Foundation, as well as Akili Interactive. None of the compensation was contingent upon any specific outcomes or findings from the research reported herein nor, for that matter, in any of EM's research publications. EM also receives compensation from the National Institutes of Health for her participation in peer grant review. RC is the executive director of Growing Healthy Children Therapy Services in Rescue, CA where she is a treating occupational therapist, conducts research, provides consultation to other therapy practices, and teaches seminars in the community. She is on faculty for the STAR Institute where she teaches courses to therapists. She is a research consultant for UCSF and UCLA. None of her compensation in the aforementioned capacities, or any capacity, was contingent upon any specific outcomes or findings from the research reported herein, nor for that matter, in any of RC's research publications. SC receives salary support as a professor of Hearing and Speech sciences and a professor of Psychiatry at Vanderbilt University School of Medicine. He is a co-developer of conversational recast intervention and phonological recast intervention which are both evidence based Naturalistic Developmental Behavioral Interventions (NDBIs). SC's research is currently supported by grants from the National Institute on Deafness and Other Communication Disorders (NIDCD) and the National Institute on Mental Health (NIMH) of the NIH, the Institute of Educational Sciences (IES) of the US Department of Education, The National Endowment for the Arts (NEA), the Scottish Rite Mason's Foundation of Nashville. The Henry Wallace Foundation provided support for the research included in this special issue. He receives royalties for two books: The Intuitive Parent (2017) Penguin/Current and Late Talking Children: A Symptom or a Stage? (2014) MIT Press and is also coauthor of the Woodcock-Camarata Articulation Battery (WCAB, 2020) Schoolhouse Publications and receives royalties for this test. SC has no direct or indirect financial interest in the results presented herein other than to declare the research support provided by the Wallace Foundation.
